# Machine learning for adaptive deep brain stimulation in Parkinson’s disease: closing the loop

**DOI:** 10.1007/s00415-023-11873-1

**Published:** 2023-08-02

**Authors:** Andreia M. Oliveira, Luis Coelho, Eduardo Carvalho, Manuel J. Ferreira-Pinto, Rui Vaz, Paulo Aguiar

**Affiliations:** 1 Faculdade de Engenharia da Universidade do Porto, Porto, Portugal; 2 Neuroengineering and Computational Neuroscience Lab, Instituto de Investigação e Inovação da Universidade do Porto, Porto, Portugal; 3 Instituto Superior de Engenharia do Porto, Porto, Portugal; 4 ICBAS–School of Medicine and Biomedical Sciences, University of Porto, Porto, Portugal; 5 Centro Hospitalar Universitário de São João, Porto, Portugal; 6 Faculdade de Medicina da Universidade do Porto, Porto, Portugal; 7 i3S-Instituto de Investigação e Inovação em Saúde, Rua Alfredo Allen, 208, 4200-135 Porto, Portugal

**Keywords:** Adaptive deep brain stimulation, Machine learning, Closed-loop control, Biomarkers, Parkinson’s disease

## Abstract

Parkinson’s disease (PD) is the second most common neurodegenerative disease bearing a severe social and economic impact. So far, there is no known disease modifying therapy and the current available treatments are symptom oriented. Deep Brain Stimulation (DBS) is established as an effective treatment for PD, however current systems lag behind today’s technological potential. Adaptive DBS, where stimulation parameters depend on the patient’s physiological state, emerges as an important step towards “smart” DBS, a strategy that enables adaptive stimulation and personalized therapy. This new strategy is facilitated by currently available neurotechnologies allowing the simultaneous monitoring of multiple signals, providing relevant physiological information. Advanced computational models and analytical methods are an important tool to explore the richness of the available data and identify signal properties to close the loop in DBS. To tackle this challenge, machine learning (ML) methods applied to DBS have gained popularity due to their ability to make good predictions in the presence of multiple variables and subtle patterns. ML based approaches are being explored at different fronts such as the identification of electrophysiological biomarkers and the development of personalized control systems, leading to effective symptom relief. In this review, we explore how ML can help overcome the challenges in the development of closed-loop DBS, particularly its role in the search for effective electrophysiology biomarkers. Promising results demonstrate ML potential for supporting a new generation of adaptive DBS, with better management of stimulation delivery, resulting in more efficient and patient-tailored treatments.

**Supplementary Information:**

The online version contains supplementary material available at 10.1007/s00415-023-11873-1.

## Introduction

Deep brain stimulation (DBS) is a therapy applied in an increasing range of neurological disorders, including Parkinson’s disease (PD). Second to Alzheimer’s disease, PD is one of the most common neurodegenerative diseases worldwide, with tremendous impact in society [1]. Currently available pharmacological treatments for PD are symptom oriented and there are no disease modifying therapies. These treatments achieve symptomatic control in a considerable proportion of patients but there is still a significant number of patients for whom pharmacologic therapies are ineffective or insufficient, leaving DBS therapy as the remaining option.

For the implementation of DBS in PD patients, an electrode is surgically placed in the brain, providing electrical stimulation patterns to a specific target area (the subthalamic nucleus (STN) or the globus pallidus *pars interna* (GPi)). In the initial setup after surgery, stimulation parameters are explored by the clinician in search for the configuration achieving the proper symptomatic control. Each parameter is tuned within a range of values where the intervention is potentially beneficial to the patient (for a brief overview of the tuneable parameters and their known effects see Table S1). Outside these subjective and patient dependent window’s boundaries, the therapy is not as effective and adverse effects might occur. Information on the precise lead location, obtained via imaging techniques, is also often taken into account in this process. The stimulation parameters are adjusted by a trial-and-error approach, relying on qualitative evaluation of clinical responses [2, 3]. The parameters are then kept fixed for the time between consultations. Given the dynamic nature of PD symptoms, this canonical open-loop stimulation strategy is suboptimal, since the applied therapy does not automatically adapt to the patient’s state nor condition evolution. Furthermore, its adverse effects (generally caused by the continuous stimulation with constant amplitude) are difficult to manage.

Progress towards optimized DBS (on both open and closed loop approaches) is challenged by the fact that there is still no clear understanding of its mechanisms of action on neuronal populations and circuits [4, 5]. While the exogenous electrical current applied by the electrode exerts direct effects on locally-residing cell bodies, which can be of inhibitory or excitatory nature, it also perturbs *en passant* axons, resulting in the modulation of complex neural networks [6–9]. Indeed, multiple mechanisms of action are likely to occur simultaneously. On one hand, this adds complexity to our understanding of the modulation process but, on the other hand, it also opens alternative potential targets to explore as to improve the neuromodulation results. Possible therapeutic mechanisms involve the direct suppression of local pathological neuronal activity, masking of pathological activity, and interactions with re-entrant nonlinear oscillations [7, 10–15].

**Fig. 1:**
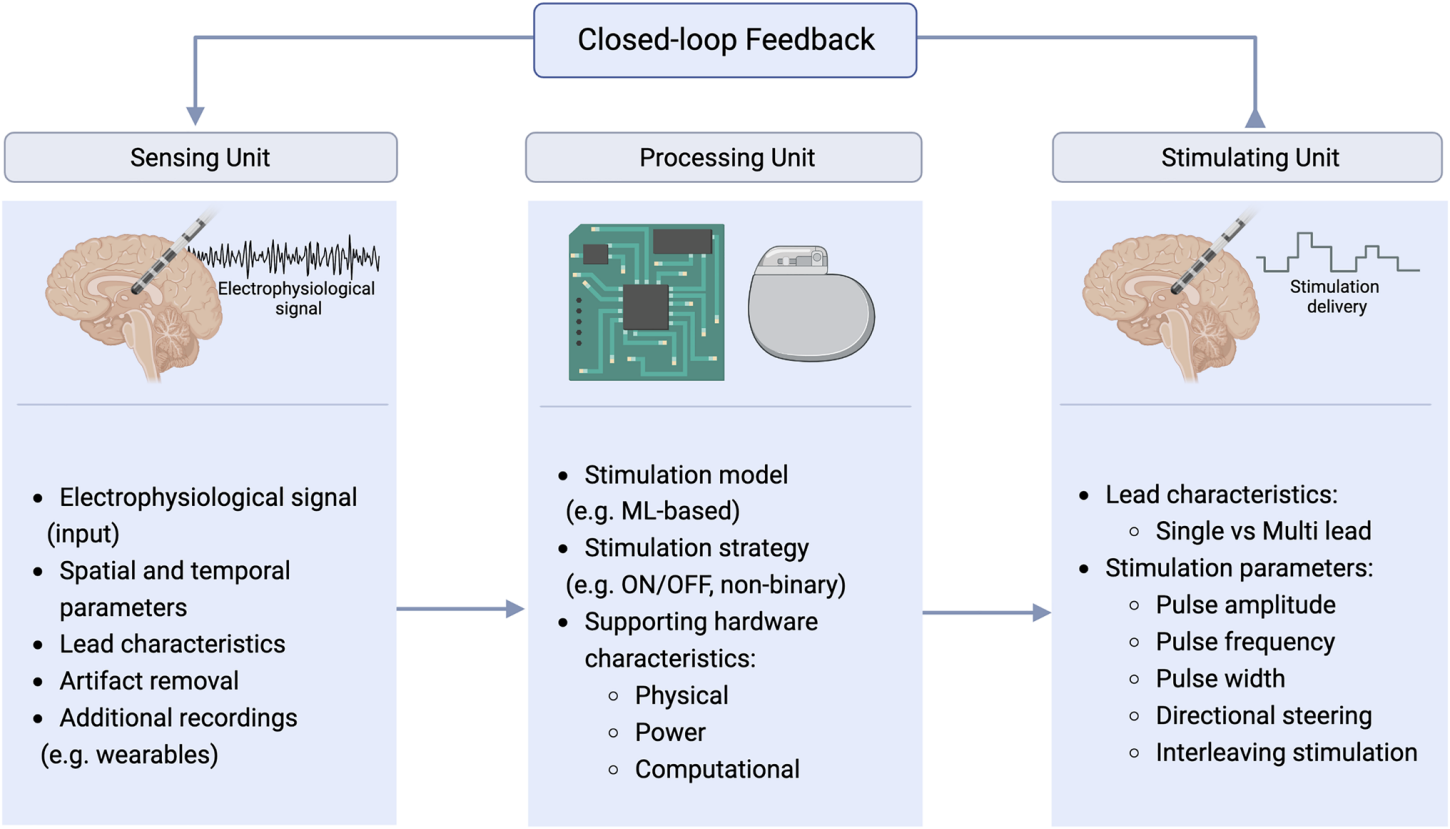
A development framework for closed-loop control supporting adaptive Deep Brain Stimulation (aDBS). The closed-loop control integrates three key components or units. The sensing unit refers to the sensing lead and signal acquisition. The processing unit refers to the stimulation strategy and model, and the supporting hardware characteristics. The stimulation model can rely on advanced algorithms based on machine learning (ML) methods. The stimulating unit refers to the stimulation parameters and the stimulating lead. For each unit, relevant associated parameters are presented

Adaptive DBS (aDBS) emerges as a promising paradigm to achieve “smart” DBS systems (Fig. 1). The principle behind this proposed approach is the optimization of stimulation parameters in direct response to the patient’s electrophysiological state, achievable in a closed-loop configuration. Closed-loop control mechanisms are associated with algorithms capable of automatically regulating the system’s parameters in order to maintain a desired/predefined target state, without human interaction. For aDBS, this means that DBS stimulation parameters continuously adjust to the patient’s varying needs. Considering the different possible strategies to close the loop in aDBS, the feedback systems directly based in neuronal signals are of particular interest. These feedback control systems become possible with real-time neuronal activity recordings obtained directly from the target region using dual function electrodes (providing concurrent stimulation and recording). Recent neurostimulators are modified to operate with these novel sensing electrodes capable of recording local field potentials (LFPs, extracellular potentials generated by the synchronized electrical activity of closely located neurons in the vicinity of an electrode [16]) together with the delivery of stimulation. This direct access to the neuronal activity domain enables the potential identification of electrophysiological biomarkers linked to pathological states of the disease (here “biomarkers” are not molecular in nature but represent instead specific signal patterns). Monitoring these biomarkers (which may be patient specific) and exploring their variability enables the adaptation of different stimulation parameters according to the needs of each patient and in response to the disease progression over time. This leads to a potential increase in the quality of life of the patient whilst lowering the cumulative effect of therapy. Unfortunately, there are still challenges that must be overcome for the successful development and implementation of electrophysiology-based closed-loop aDBS. A significant part of these challenges is related to the complex signal analysis and to the stimulation modulation.

Machine Learning (ML) methods are rapidly growing in popularity as powerful computational tools to deal with the complex data from DBS and pave the way to “smart” systems. These methods can give computers the ability to learn and improve from accumulated data (“experience”), without the need to define algorithms or specific dependencies between variables. ML offers methods to explore complex structure and patterns in the data, that are not easily identifiable with traditional methods. Given its versatility, ML can be useful at different stages of the DBS therapy process such as patient selection [17, 18], electrode placement during surgery [19–21], prediction of outcome [22–24], and management of stimulation [25]. The latter is linked to the identification of discriminatory electrophysiology biomarkers and associated modulation of stimulation parameters, crucial to the development of personalized and adaptive control systems for an effective symptoms relief.

The application of ML towards the development of components of an aDBS system based on electrophysiology biomarkers is the focus of this review. We discuss the current strategies and challenges towards these “smart” closed-loop aDBS technologies, and explore how ML can contribute to their development. In specific, we draw our attention to the use of ML for the identification and validation of biomarkers. PubMed was used for the literature analysis, focusing on publications with date ranging from 2015 to 2023. Further information regarding the included papers, such as the methods used and its contextualization in the DBS therapy, can be found in Tables S2–S6. In the next section, we start by providing the core concepts and terminology of ML. We then move on to exploring the search for effective biomarkers, providing fertile ground to discuss different aDBS strategies and technologies. We close the review with the discussion of the road ahead for achieving aDBS with the application of ML.

## Core concepts in machine learning

Machine learning methods are a large family of data-driven algorithms capable of automatically improving their ability to make decisions/predictions through accumulated data exposure (“experience”). The application of a ML method often implies training a model with the available data, which is then tested and validated according to given performance criteria. Different metrics (defined according to the problem) are used to evaluate the performance of the model and select the most adequate for the task. There are three main types of methods used in machine learning. In *supervised learning*, the models are trained on annotated datasets (with labelled input–output correspondence). In *unsupervised learning*, methods look for hidden patterns or internal structures in the input, without pre-defined information about the output. Finally, in *reinforcement learning* a computational agent is trained based on policies (“rules”) that reward desired actions and punish unwanted ones. Whereas reinforcement learning methods yield a relevant potential for training neuromodulation systems, they have not yet been employed in the context of DBS for PD (but see [26]). As such, in this review we focus on unsupervised and supervised learning methods successfully used in DBS.

Unsupervised learning algorithms can help in dimensionality reduction (search for a reduced set of variables describing the data), feature extraction (identifying which variables can be helpful for discrimination and which are redundant), and clustering (grouping sets of data according to similarities or goals). Such algorithms can be used per se, to support decisions or, in combination with data representation techniques, to gain new insights (e.g. the number of classes contained in the data, how the data is distributed, and what patterns exist within the data) leading to a better selection of models in a subsequent processing stage (Fig. S1).

**Fig. 2:**
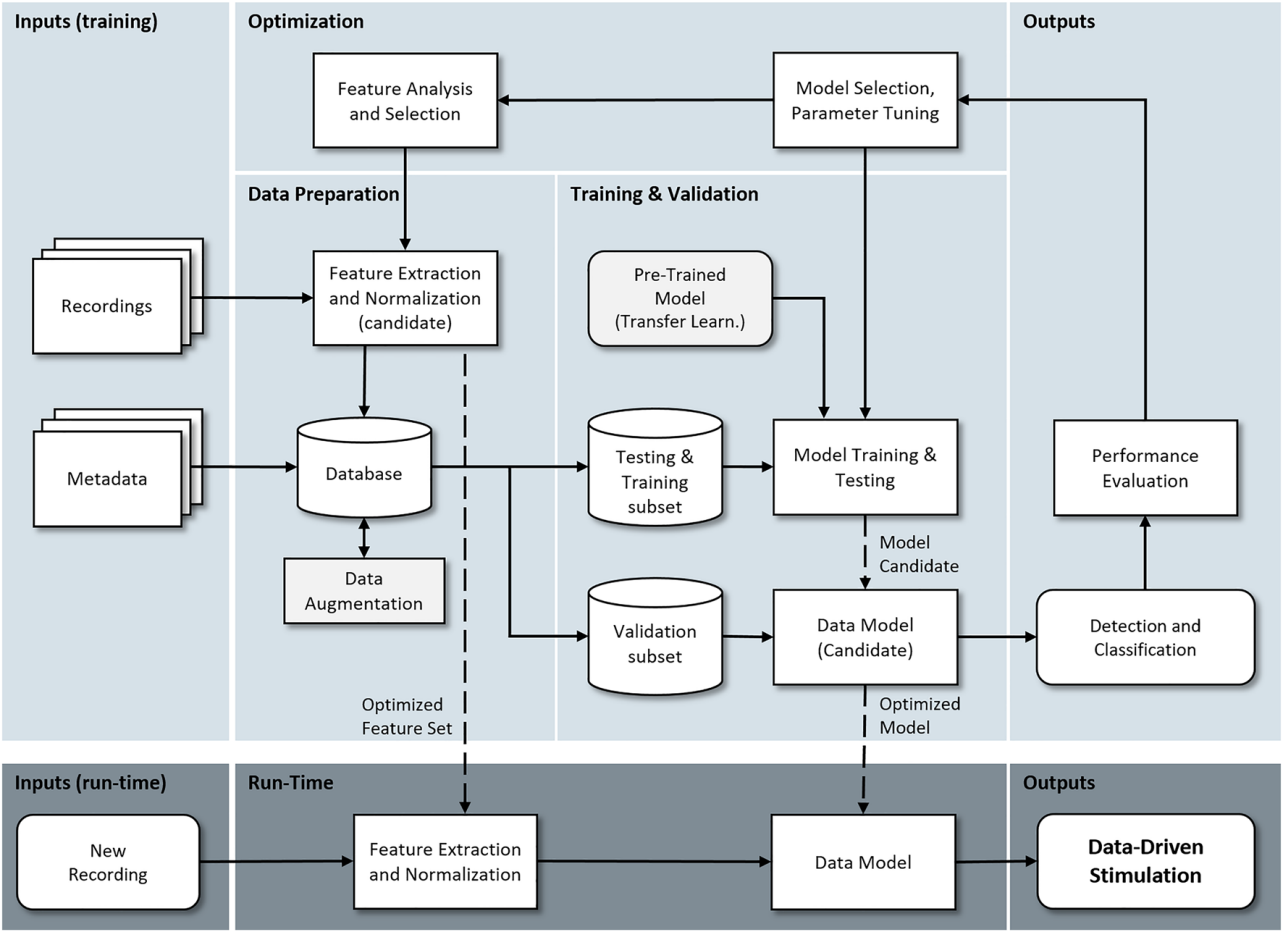
Generic workflow associated with the application of supervised learning methods to achieve data-driven stimulation. In light grey (top) is the development pipeline and in dark grey (below) is the deployment pipeline

Supervised learning algorithms require labelled data (for every input there must be a matching annotated output) and two data subsets should be defined: a training subset, for adjusting the model’s parameters; and a testing subset, for model evaluation with data that was unseen during training (a validation subset may also be considered for adjusting the model’s hyper-parameters). This division in training data and testing data is fundamental and addresses the ability of the model to *generalize*, i.e. preserve adequate performance with new non-annotated data. As real-world data is noisy (from both instrumental and physiological sources), there is the risk of attaining high performance levels in the training data but low levels in the new data (a situation called *overfitting*). Concerning the outputs (predictions) in supervised learning, so called regression methods produce continuous values whereas classification methods are associated with categorical/discrete outputs (Fig. S1). A generic workflow to support the development of classification (and detection) methods in the context of data-driven stimulation is presented in Fig. 2.

A particular ML method called deep learning has gained considerable relevance, due to its ability to provide good estimates even when facing unstructured high dimensional data. This method is inspired by the dynamics of biological neurons and uses nodes (neuron analogues) which are combined in large architectures with several sequential layers to provide the output. The number of elements per layer, the number of layers, and the behavior of each layer (e.g., fully connected, convolutional, recurrent) are some of the parameters that can be adjusted to fit the network to the data/problem. Despite the widespread use of these techniques, the high amount of data that is required for training and the “black-box” model that is obtained in the end are strong caveats of deep neural networks. Nevertheless, visualization tools, data augmentation and transfer learning are techniques that can help solving these issues.

Both supervised and unsupervised ML methods, in a variety of different algorithms, have already been effectively used with DBS data (Table 1). In all these methods, the quality of the data deserves special attention: incorrect or poor-quality data (e.g., outliers, wrong labels, noise), if not properly cared for, will lead to under-optimized models and to unsatisfactory results. In the ML context, the discussion is often focused on the decision of the model and on the performance metrics to be considered during the development phase. In a clinical setting, the relative impact between false positive predictions and false negative predictions is an important issue on the choice of performance metrics.

**Table 1:** Machine learning methods in DBS

Type/purpose	Method name
Unsupervised	
Clustering	Gaussian mixture model K-means
Dimensionality reduction/feature selection	Linear discriminant analysis (LDA) Principal component analysis (PCA): kernel PCA t-distributed stochastic neighbour embedding (t-SNE)
Supervised	
Classification	AdaBoost Decision Tree (DT): Oblique DT Gradient Boosting Machine: XGBM, Extreme gradient boosted trees Hidden Markov Model (HMM) K-nearest neighbor (KNN) Logistic Regression (LR): L1 logistic / LASSO; L2 logistic/Ridge Naïve Bayes (NB): Conditional model and Gaussian Neural Networks (NN): Multilayer perceptron, Shallow NN, Convolutional NN (CNN), Deep NN, LAMSTAR NN, Recurrent Networks Random Forest (RF): unsupervised RF Support-vector machine (SVM): SVM based on linear and Radial Basis Function (RBF) kernels
Regression/time series	Granger causality Linear regression Kalman filters Recurrent networks Volterra kernels

The methods are organized according to the following categories: Clustering (Ct) and Dimensionality Reduction/Feature Selection (DR/FS) for unsupervised learning; Classification (Cf) and Regression/Time Series (R/TS) for supervised learning. It should be noticed that some methods can be adapted to operate with different purposes. For example, recurrent networks can be used either in a context of classification or regression

Since these approaches are "data-driven", it is essential to ensure the quality of the supporting data, used in the training phase, and to monitor the operation, in the deployment phase. Training datasets should be sufficiently diverse to guarantee that a wide range of operational situations are anticipated. The amount of data should be sufficient to ensure a reasonable fit of the model parameters, and should be balanced across classes, so that trends or decision bias do not arise. Regarding the operational phase, it is important to guarantee that safety limits are established, and that regulatory and contingency mechanisms are foreseen.

## The quest for effective biomarkers

The concept of biomarkers plays a central role in the development of feedback control systems for aDBS. A biomarker refers to a characteristic in a physiological signal, such as a specific pattern or component, which correlates to a certain pathological state. Different motor or electrophysiological signals, such as electroencephalography (EEG) signals and LFPs, have shown potential for biomarker identification, and consequently closing the loop in aDBS [3, 27–34]. LFPs present interesting advantages over EEG due to the source location and data fidelity, a possibility given the now available electrodes with combined stimulation and sensing properties, and appropriate long-term stability at the electrode-tissue interface [35]. Different neurological conditions have already been studied in the context of closed-loop DBS systems using LFPs as biomarkers (for an overview, see [36]).

Several aspects come into play in the identification of effective biomarkers and their selection is determined by the disease context and symptoms manifestation. Key features to consider during the selection of either single- or multi-dimensional biomarkers are the high signal-to-noise ratio of the recordings and the stability in the presence of external artifacts. These artifacts can be caused by non-pathological functions, such as intentional movement. In fact, one issue with the use of beta-band subcortical oscillations as a biomarker in PD is that they are also involved in the modulation of voluntary movement [36]. Naturally, important features also include the sensitivity and the specificity of the biomarkers, to ensure the correlation in time with the severity of the clinical symptoms [37, 38]. Independently of the source/type of the physiological signal, the search for strong correlations between signal features and pathological states is a challenging analytical task, in which ML methods can have an important contribution.

### Motor biomarkers

With motor symptoms being a hallmark of PD, several studies have focused on assessing strong correlations between motor features and clinical states. Given that motility is an intrinsic feature of the human experience, it is crucial to understand what constitutes a pathological motor pattern and how it differs from normal motor activity, be it volitional or not. A common source for the study of motor data are the so called wearables: sensors that can be worn and provide detailed motor/physiological information about the patient [39]. This objective information serves as a valuable addition to the clinical data when assessing symptoms and their response to stimulation, enhancing confidence in the chosen stimulation parameters. By providing objective measurements, it also alleviates the workload on the clinicians. One of the major advantages of wearables is that they can be easily integrated in ordinary devices, such as smartwatches or rings. For instance, Powers et al. developed a motor fluctuations monitor for Parkinson’s disease algorithm, integrating long term data collection from smartwatches, capable of evaluating tremor severity and the presence of dyskinesia [40]. Additional information on movement (e.g. extracted from smartphones) can also emerge from crowd-generated data as a proxy for wearables, as shown by Zhang et al. In their work, convolutional neuronal networks (CNN) and random forest (RF) applied to smartphone accelerometer and gyroscope recordings were used to differentiate PD from control subjects [41]. Alternatively, Kleinholdermann et al. explored the use of non-invasive movement recordings to predict the best DBS settings [42]. In their work, RF models were trained to learn the relation between electrode settings, clinical ratings, and movement features (extracted from wearables). Their work shows not only that movement features were able to predict the clinical mobility (correlations up to *r = *0.68 between the predicted and true values), but also that these features predicted the optimal DBS parameters (*r = *0.8).

Discrimination between PD and healthy subjects is also possible using information from wearables placed on a single arm with the implementation of the K-nearest neighbors (KNN) algorithm, providing a quantitative assessment of bradykinesia, rigidity and tremor [43]. Moreover RF algorithms using 3D gait data and motor readout signals have been used to show that a standing up test can be used to distinguish PD OFF DBS patients from healthy subjects; also, foot and lower leg kinematics are better in classifying motor anomalies than other gait analysis segments [44, 45]. This contributes not only to the diagnosis of PD, but also to the monitoring of the symptom progression and response to treatment. Different approaches for monitoring symptoms, combining sensors for motion, muscle activity and force, were presented by Angeles et al. and Tahafchi et al. [46, 47]. These studies used, respectively, K-nearest neighbors and support vector machines methods to predict motor symptom severity and Freezing of Gait (FoG) events. Liu et al. took advantage of yet another RF model to classify FoG states based on the Codamotion 3-D movement capture system and synchronized intracranial EEG data, further broadening the understanding of movement dynamics and contributing to bridging the gap between peripheral and intracranial signals [48].

It is important to recall that DBS is not a substitute to pharmacologic treatment, but rather a complementary therapy. Consequently, monitoring response to medication should also be considered when designing aDBS systems. In this respect, Khodakarami et al. applied linear regression models to predict the motor symptom state based on the response to the first morning dose of levodopa [49].

The ability to detect and analyze motor patterns (directly or indirectly) may turn out to be an indispensable component of aDBS systems, capable of adjusting function over different time horizons and symptom intensities. Yet, one should not forget that the symptoms measured by wearables are but a peripheral manifestation of a neurological condition. Furthermore, the location of the wearable will affect the extracted features, and there is not a consensus on the best approach for these measurements. The information of wearables alone is, most likely, not sufficient to fulfil the needs of aDBS implementation: some of the neurological traits may not translate into noticeable motor manifestations and have non-motor implications on the patients.

### Electrophysiology biomarkers

Electrophysiology signals coming from the stimulated neuronal populations can be explored either in the time domain (amplitude as a function of time) or in the frequency domain (decomposition in its frequency components; Fig. S2). The latter, often studied using spectral analysis, is generally preferred not only because it facilitates both noise and artifact identification and reduction, but also because it allows the decomposition of the signal in pre-defined frequency bands (Table S7) [37]. Variations in the power of these bands have been associated (correlated) with different PD symptoms, showing their potential to work as biomarkers for pathologic events [38]. However, accounting for the complexity of neuronal dynamics, reducing the biomarker to a frequency band’s power may leave out useful information [50]. Here, ML can help to uncover patterns that traditional signal processing tools do not.

Electrophysiology datasets (and LPFs datasets in particular) are often small, structured, and noisy. Consequently, specific techniques have been developed to circumvent these challenges. For example, these datasets are amenable to gradient-boosted tree learning, a popular ML method for prediction. Hirschmann et al. effectively used this method to demonstrate that neuronal oscillations in LFPs provide accurate predictions of motor symptom improvements following DBS [51]. In addition to signal band power, the signal coherence between subthalamic and parietal regions was shown to have relevant predictive power of DBS’ therapeutic efficacy. With their approach, it was possible to study not only the predictive capability of the LFPs, but also which features would contribute more to a potential clinical application (among the best being the sub-thalamic high beta power). Additionally, logistic regression was able to identify periods when patients have rest tremor using LFPs from the subthalamic nucleus [52]. Two studies with LFPs, using neuronal networks and hidden Markov model classifiers were also successful in decoding movement and laterality (the side in motion), as well as tremor prediction [53, 54].

Another approach to classification of human behavior with LFPs signals and considering five tasks (speech, finger movement, mouth movement, arm movement, and random segments), was possible using support vector machines (SVM) [55]. In Golshan et al., more complex methods such as CNNs were used to classify the human behavior using the time–frequency representation of STN-LFPs within the beta frequency range [56]. Interestingly, even simpler classification methods, such as the naïve Bayes classifier, can extract valuable information from LFPs. For example, LFPs dynamics on specific frequency bands (alpha and beta) allow accurate prediction of intentional limb movements, ahead of their execution [57]. Further combinations of ML techniques with Kalman filters applied to LFPs allowed the identification of biomarkers such as the power in the high beta frequency band, high gamma frequency band, and low beta frequency band for tremor detection [58].

It is also worth mentioning that potential biomarkers can be explored in a variety of neuronal activity metrics. This includes synchronization levels and long-term correlations between neuronal signals from different sources (e.g., different hemispheres or intracerebral/peripheral). For example, SVMs algorithms and Gaussian Naïve Bayes models have been applied to LFPs recordings to decode movement behavior using frequency dependent neural synchronization and inter-hemispheric connectivity features [59]. Recent literature emerged on the value of evoked resonant neural activity (ERNA) signals as potential biomarkers, however, its use is still under validation [33, 34].

Finally, it should be highlighted that the search for personalized biomarkers is gaining some traction. This is not surprising, given that the disease manifests and evolves differently in each patient. The “one size fits all” approach is being replaced by tailoring the treatment according to individual needs. Mohammed et al. implemented patient specific features extraction in combination with adaptive SVM classifiers [60]. The outcome of this method provided individualized features and a classifier for each patient. This highlights the importance to account for the heterogeneity of symptoms across patients and tones down the expectation on finding reliable population-wide neural biomarkers. On a similar note, supervised methods were used for the identification of individual neural biomarkers of hand motor performance in PD patients undergoing DBS. In this study, patient specific biomarkers improved decoding accuracy compared to group-level [61].

### Other biomarkers

Besides affecting motor control, PD is also characterized by a myriad of non-motor symptoms, such as speech alteration and insomnia, highly debilitating and often hard to diagnose and manage. These symptoms could also, in principle, support relevant biomarkers. However, the identification and monitoring of non-motor symptoms poses serious challenges, due to their subjective nature. Matters become more complex if one intends to correlate these symptoms with intracranial electrophysiology signals from sensing electrodes. Regarding non-motor symptoms that can be monitored, two groups are highlighted here: speech and voice alterations, and sleep and cognition.

Speech and voice features can provide additional digital biomarkers as well as valuable information for the screening of PD patients. Given the complexity of these signals, ML methods have also been used for this type of analysis. In Braga et al., speech recordings in uncontrolled background conditions were used to identify PD patients and to estimate early signs of PD using ML regression models [62]. On another approach, by extracting paralinguistic features of voice recordings, Tracy et al. explored the possibility to predict PD severity, using both classification and regression methods. However, no clinical validation was provided in the study [63].

Within non-motor symptoms, both sleep quality and cognition worsen with progressing PD. They are particularly challenging to address, even when using ML algorithms since the collected data has a strong level of subjectivity inherent to the available assessment tools [64]. However, the study of electroencephalographic recordings shows potential to extract biomarkers for post-operative cognitive decline deterioration [65, 66]. Using LFPs recordings during wakefulness and nocturnal polysomnography sleep as inputs to SVMs and decision tree algorithms, Chen et al. showed that it is possible to classify sleep stages in PD [67].

Clinical information (admission details and UPDRS-III evaluations) has proven to be helpful in the prediction of PD progression and its response to therapy. For example, Khojandi et al. have used preoperative PD rating scores and clinical characteristics to feed a RF algorithm to predict the appropriate DBS stimulation frequency (either 60 Hz or 130–185 Hz) for a given patient [68]. In the context of PD but not directly related to DBS, Shamir et al. present a proof-of-concept of a clinical decision support system that applies ML techniques such as SVMs and RF to clinical data in order to assist in treatment management [69].

All things considered, biomarkers with high specificity and sensitivity will have a crucial role in the multiple stages of PD management: either as immediate sources of clinical information or as the means for more effective DBS protocols. Wearables, and other sources of peripheral signals related to PD symptoms, are an important strategy to provide information on the patient’s clinical state. However, the use of additional external hardware can be uncomfortable for the patient and unnecessary/redundant if neuronal (electrophysiology) biomarkers can provide equivalent information. ML techniques have been successfully applied in the study of the PD symptomatology, especially in intracranial electrophysiological signals. The ideal closed-loop control systems would probably rely solely on intracranial signals, and, in this sense, the use of LFPs related signals, captured by modern DBS systems, may be the best solution. LFP analysis should consider not only isolated values of the parameters (single or combined) but also their long-term temporal evolution and intrinsic patterns, both in physiological and pathological states. These provide a better understanding of the patient’s state, leading to the design of better control strategies. A clinically validated classification system based solely on intracranial signals has not yet been achieved, but the first steps have been taken. Although early DBS literature aimed towards the finding of generalized biomarkers for PD, recent works are increasingly adopting a personalized framework, giving primacy to individual-specific biomarkers.

## Bringing adaptive DBS to the clinic

Adaptive DBS systems must simultaneously monitor (record) physiological signals and provide stimulation to the neuronal targets, while adjusting the stimulation parameters according to disease specific biomarkers reflecting the current state of the patient. It is worth mentioning that the neuronal tissue response to electrical stimulation may fluctuate over time as a result of external (environmental) or internal (disease progression) factors [70]. Efficacy of aDBS is further challenged by neuronal plasticity, which is thought to contribute to the loss of therapeutic effect over time in an open-loop framework.

**Fig. 4:**
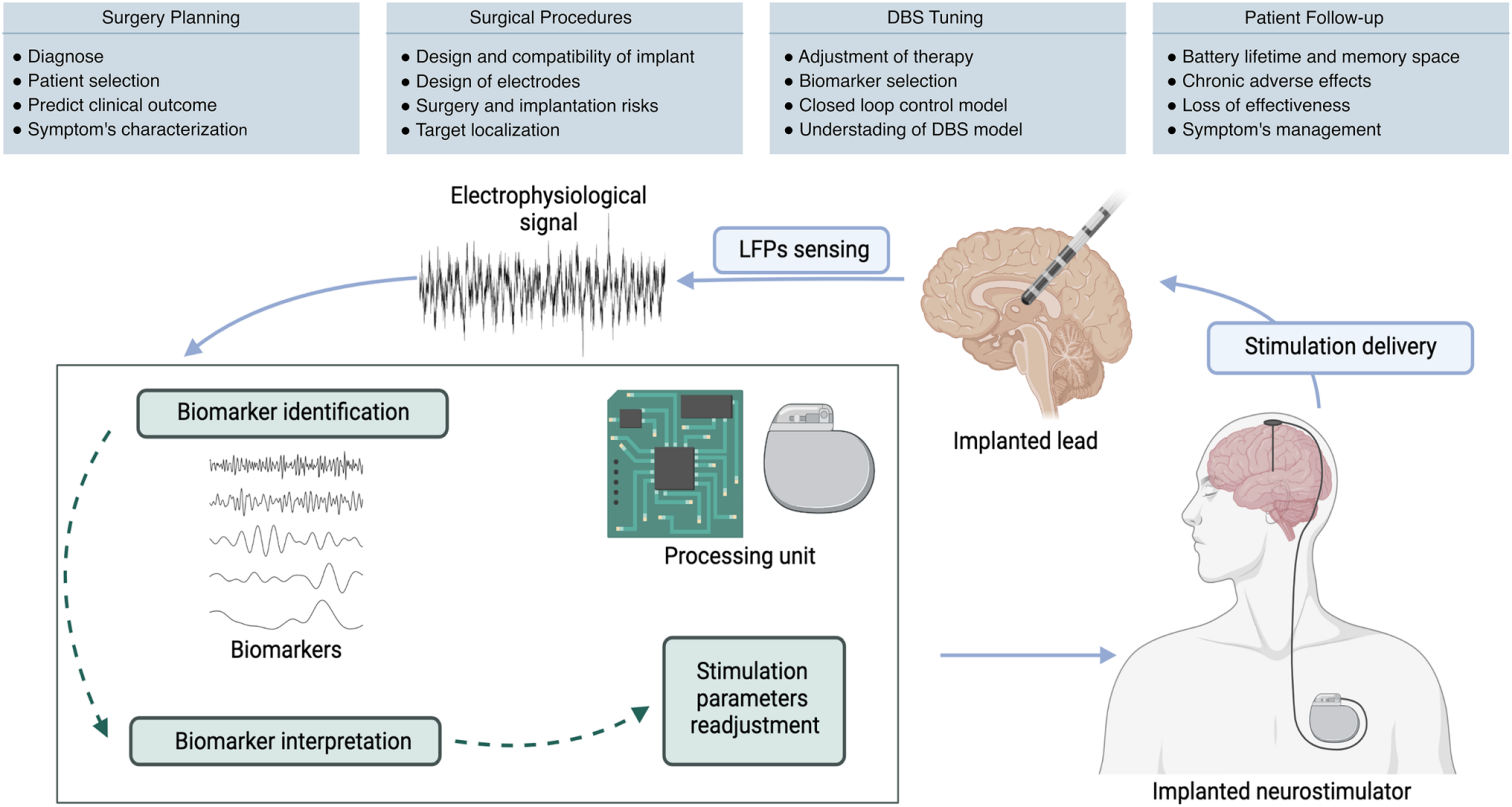
Application of machine learning towards adaptive deep brain stimulation using closed-loop control. In the top panel, a list of challenges at different stages of DBS therapy implementation where ML methods can play an important role. The diagram represents a closed-loop feedback system for aDBS, based on LFPs sensing and electrophysiological biomarkers identification and interpretation

Three main components are required for an aDBS system with closed-loop control using LFPs biomarkers (Fig. 4): the neurostimulator (controllable pulse generator) that provides the stimulation, a lead that delivers the stimulation and simultaneously records the LFPs, and a processing unit that reads and interprets the biomarkers present in the LFPs and decides the appropriate stimulation response. Recent technological advances in the neurostimulation devices expanded the range of tunable parameters beyond the typical pulse parameters (amplitude, frequency, and pulse-width). For example, with the availability of new leads with directional stimulation capabilities, it is also possible to shape the electrical field used in DBS [4, 71–74]. All these parameters can be, a priori, usable by ML algorithms for closed-loop aDBS.

### Adaptive DBS control strategies

Adaptive DBS requires Implantable Pulse Generators (IPGs) with signal sensing and recording capabilities, in addition to the stimulation delivery, in order to conduct biomarkers monitoring and generate the stimulation protocols in a real-time manner [75, 76]. Biomarkers are not required to be causally linked to the disease mechanisms, but rather to show strong correlation with symptom onset and severity, and to be sensitive or adaptive to the disease progression [77–80]. Nevertheless, a causal relation would be ideal since it allows the identification of changes prior to the onset of symptoms, enabling a predictive approach rather than a responsive one. A common strategy in neuromodulation that employs preventive (and predicted) measures is the delayed feedback control method, entailing closed-loop techniques designed to desynchronize abnormal neuronal activity. Aiming at the suppression of pathological collective synchrony, effective and robust desynchronization of STN GPi model neurons has been shown by stimulation protocols based on delayed feedback control [81, 82]. The latency of the response is also an important factor to account for. Current neurotechnologies allow for virtually instantaneous and continuous adaptation of the stimulation parameter to the monitored biomarkers [83]. However, it is yet not clear how refined should the temporal resolution for aDBS be, considering the optimization of therapeutic effect and the parallel computational requirements. In Rosa et al., stimulation intensity was linearly adjusted every second, in proportion to LFP beta power. Despite only being deployed in one patient, this approach improved symptom control [84]. In Little et al., ON/OFF stimulation was adjusted continuously in a closed-loop system with improvements on motor symptoms in an eight-patient cohort [83].

#### Amplitude modulation

Amplitude is one of the pulse parameters that can be used to modulate the stimulation in aDBS systems [83, 85, 86] (Fig. S3). The simplest paradigm consists in the delivery of stimulation with a predefined non-null amplitude only when necessary (ON/OFF controller). Other paradigms modulate the amplitude in different ways: gradually (or, step-wise) and continuously [87]. During gradual amplitude modulation, the amplitude is maintained between two defined discrete values and varies in a step-like fashion [32, 88]. In continuous modulation, the amplitude of the stimulation varies (proportionally, in general) according to a reference signal/biomarker. The output amplitude reflects, with minimal delay, the alterations of the input signal [75, 84, 89].

#### Frequency and pulse-width modulation

Changes in the frequency and pulse duration of the stimulation have an impact on the neuronal response and can be used in a closed-loop context. Although the involved biological mechanisms remain unclear, high-frequency stimulation (> 100 Hz) generally produces better therapeutic results than low-frequency stimulation [90, 91]. The initial parameters for frequency and pulse-width are often in the ranges of 130–180 Hz and 60–90 µs, respectively. The fact that different symptoms respond better to specific frequency intervals is taken into account when setting parameterizations for personalized treatments [92]. Recent studies have explored the use of shorter pulse widths, which appear to offer comparable therapeutic effects with better energy management and reduced adverse effects [93]. Changes in the pulse-width have also been shown to provide clinical benefits by exciting brain sites more selectively [93, 94].

#### Phase modulation

A core concept supporting phase-based aDBS is the hypothesis that the precise timing of stimulation is important for its effectiveness. This is particularly relevant in the context of trying to suppress pathological collective synchronization across neuronal populations. In this approach, the stimulation signal is derived from the phase response curve of the monitored neuronal population. Under this hypothesis, adapting the stimulation at the timing that most effectively suppresses the patients tremor, appears to reduce side effects and the amount of stimulation delivered, without compromising clinical results [81, 82, 95, 96].

Concluding, one should emphasize that pioneering studies using ML to support real-time data analysis in closed-loop control strategies for aBDS in PD have already started to emerge. In Gilron et al., subcortical and cortical oscillatory signatures of motor state were used as control signals to achieve a fully embedded adaptive DBS, working outside clinical settings. Both supervised and unsupervised ML methods were implemented to assess the possibility for using (i) oscillatory phenomena in PD to decode motor fluctuations; and (ii) multiple recording sites to improve the classification of an individual’s motor state [97, 98]. In other movement disorders, such as essential tremor, ML-based stimulation protocols were already successfully applied [25]. Notwithstanding, two main concerns persist. The first has to do with the computational cost of some of these algorithms. Given the power limitations of the current processing, aDBS is advised to operate on low complexity algorithms. Secondly, the development of such system will require progressive iteration, starting from works such as the one present by Gilron et al., and learning from the protocols applied to other pathologies.

## Open challenges in aDBS–future roadmap

As already explored, one of the major challenges of aDBS is the identification and validation of reliable biomarkers with high specificity and sensitivity. The recent developments in sensing technology widens the opportunity to understand how the electrophysiology at the site of stimulation evolves with the pathology. In this regard, LFPs from neurons in the stimulated regions are a prime candidate for the identification of biomarkers. For better analysis of the electrophysiological signal, novel techniques for artifact removal are important to improve the robustness of LFP-based biomarkers [99, 100]. In the established literature, attention has been devoted to the LFPs’ beta band power but, as addressed earlier, other frequency bands such as alpha and gamma also convey valuable information regarding the patient’s clinical state. Representations outside the frequency domain should also be better explored, as features in the time domain may prove useful for defining biomarkers.

Furthermore, the variability on disease and symptoms across patients motivated an increased attention on personalized therapies, where individualized biomarkers and algorithms incorporating learning capabilities are necessary. To have maximum efficacy, “smart” DBS is required to act as a personalized medicine approach. Since “smart” controllers require a training stage (either to learn from scratch or adapt), one solution is to have a period in which each patient undergoes an initial calibration for aDBS therapy, where the control strategy parameters are tuned [87]. Another factor to take into account is the importance of having a control system that is able to react to patients’ everyday actions and behaviors, automatically differentiating normal patterns from pathological activity. For both these requirements, machine learning emerges as a powerful tool to discriminate the (pathological) signatures specific for each patient, a feature hardly achievable by other methods. Still, clinical expertise remains as an irreplaceable component, especially during the initial parameterization and tuning of DBS, as symptoms exhibit rich and dynamic symptomatology in time scales that range from minutes to days/months.

Machine learning is already promoting advances in DBS [101]. However, ML methods also have limitations and constraints that should be kept in mind. Some classes of methods require large datasets and high-performance hardware for training the models, and the resulting black-box models are still met with caution. Moreover, the labelled datasets are often established in highly specific contexts, which limits the potential generalization of the extracted features. ML’s need for high quantity/high quality data can be met with sharing open and curated databases, allowing the replication and the exploration of published results in a collaborative scientific effort. In order to evaluate and compare the existing methods across studies, a consensus on the parameters by which performance is measured is needed. There is also a lack of long-term recorded data, hindering our knowledge on the long-term loss of therapeutic efficacy and rise of adverse effects. Moreover, the current computational requirements for closed-loop systems (model training, deployment, and algorithm implementation), may delay its implementation in IPGs for real-time therapy management.

The hardware requirements for closed-loop DBS running ML methods also raise attention as the novel computational features on these neurostimulators can add to the size and weight of the implant. Another challenge is the heat signature and energy consumption under their continuous activity, covering data acquisition, processing, and stimulation. Having a low power consumption balanced with rechargeable battery capacity and increased memory storage are requirements of future devices [4, 102, 103]. Currently, researchers are investigating electronic alternatives, such as memristors and other neuromorphic devices, aiming to achieve low-power, small-sized, and computationally efficient implantable devices [104]. Another comment goes to the importance of developing simulation environments and biophysical models of closed-loop stimulation to design and test aDBS systems before their implementation in vivo [105, 106].

Lastly, as ML becomes increasingly integrated into clinical practice, with aDBS being just one example, it is important to assess the impact of these methodologies on the clinical workflow. On one hand, the use of ML in therapies enhances the concept of personalized medicine but a clear understanding of these technologies is not integrated into clinical training (as it primarily falls within the realm of computational fields). By fostering a multidisciplinary environment, combining clinical and computational expertise, collaborative efforts can be made towards the effective development of aDBS. Moreover, as these technologies scale up, continuous data management and algorithm tuning become crucial, highlighting the need for a strong cooperation between clinical, engineering, and computational sciences.

To progress towards adaptive DBS, we must conceptualize it from the start as a multifactorial system. The challenges in this field are many, from hardware constraints to software needs, requirements of high amounts of data and the development of personalized models with clinical validation. Nevertheless, the technology has shown promising results and the associated improvements in the patients’ quality of life should encourage the device manufacturers to continue pushing for aDBS systems.

## Conclusion

This work reviews the available research on ML implementation towards aDBS in PD. Its implementation is possible throughout all the stages of DBS therapy, and in particular towards biomarker identification/selection—that may be patient-specific—and to post-operative DBS programming, using biomarker levels and adjusting a large number of stimulation parameters. The latter may particularly benefit from ML application, due to its ability to perform complex mappings between stimulation parameters and biomarkers information. Nevertheless, there are still many open challenges that need to be addressed regarding the use of ML on aDBS. Large datasets and long-term studies, carefully planned and collected, are needed for accurate model development. The application of ML extends to the IPG hardware management whose battery duration, memory space and computational power may also benefit from its use. A key challenge is the pressing need for a better fundamental understanding of PD and of the DBS physiological mechanisms. Although ML methods provide powerful descriptive models to deal with complex data and systems, alone they are not sufficient to obtain the optimal aDBS system, presenting the need for a multidisciplinary effort, covering computational and clinical sciences. Nevertheless, these technologies have shown potential to improve patients’ quality of life. This fact alone should encourage the investment in future aDBS, not only for PD but for other neurologic disorders.

### Supplementary Information

Below is the link to the electronic supplementary material.

Supplementary file1 (DOCX 566 KB)
