# Supplementary material for: Machine learning for adaptive deep brain stimulation in Parkinson’s disease: closing the loop
Source: J Neurol. 2023 Aug 2;270(11):5313–26. doi: 10.1007/s00415-023-11873-1 (PMC10576725; doi:10.1007/s00415-023-11873-1)
Supplement: Supplementary file 1 — Supplementary file1 (DOCX 566 KB) [file 415_2023_11873_MOESM1_ESM.docx]

# Supplementary Material

Machine Learning for adaptive Deep Brain Stimulation in Parkinson’s Disease: Closing the Loop

Andreia M. Oliveira^1,2^, Luis Coelho^3^, Eduardo Carvalho^2,4^, Manuel J. Ferreira-Pinto^5,6^, Rui Vaz^5,6^, Paulo Aguiar^1,2,6*^

^1^ Faculdade de Engenharia da Universidade do Porto, Portugal

^2^ Neuroengineering and Computational Neuroscience Lab, Instituto de Investigação e Inovação da Universidade do Porto, Portugal

^3^ Instituto Superior de Engenharia do Porto, Portugal

^4^ ICBAS – School of Medicine and Biomedical Sciences – University of Porto, Portugal

^5^ Centro Hospitalar Universitário de São João, Porto, Portugal

^6^ Faculdade de Medicina da Universidade do Porto, Portugal

*Corresponding Author:

Paulo Aguiar

[pauloaguiar@i3s.up.pt](mailto:pauloaguiar@i3s.up.pt)

i3S - Instituto de Investigação e Inovação em Saúde

Rua Alfredo Allen, 208; 4200-135 Porto; Portugal

**TABLE S1**. Tuneable Deep Brain Stimulation (DBS) parameters. In this case, we are considering as target the subthalamic nucleus (STN) nucleus, but the same parameters can be applied to DBS with different target regions.

| **Parameter** | **Typical ranges and observations** |
| --- | --- |
| Pulse amplitude | Most titrated parameter in the initial stages of STN DBS programming. Variation of 0.1-0.5V or 0.1-0.5mA are known to produce visible results. |
| Pulse frequency | Frequencies of 130Hz are often used as a starting point. Improvements in cardinal motor signs were shown with increasing frequency. |
| Pulse width | Conventional pulse widths used in STN DBS range from 60 to 450μs duration. |
| Directional steering | Maximizes the therapeutic response while minimizing adverse effects in other regions not relevant for the therapy management. |
| Interleaving simulation mode | Allows delivering stimulation with different energies to neighbouring anatomical structures. |

TABLES S2-S6 - Publications included in this review, organized according to the treatment stage in DBS. Concerning the machine learning algorithms, the methods applied are mentioned as well as which model group they belong to, and the type of input data used. Acronyms: CT - Computerised Tomography; EEG - Electroencephalogram; EMG - Electromyography; LFP - Local Field Potentials; qEEG - Quantitative Electroencephalogram; MER - Microelectrode Arrays; MIR - Magnetic Resonance Imaging. Model Group: Cf - Classification; Ct - Clustering; DR/FS - Dimensionality Reduction/ Feature Selection. R/TS - Regression/ Time Series.

**TABLE S2.** Publications included in the review under the category “DBS therapy management”.

| **DBS therapy management** | | | | | |
| --- | --- | --- | --- | --- | --- |
| **Author & Year** | **Stage** | **Goal** | **Input/Type of data** | **Model Group** | **Model used** |
| Castano-Candamil et al, 2019 | Planning | Propose of a fine motor function assessment framework for capturing transient DBS-induced changes. | Copy-drawn test | DR/FS | LDA |
| Boutet et al, 2021 | Release | Examine if machine learning could predict the benefits in specific Parkinsonian signs when informed by precise locations of stimulation. | Unified Parkinson’s Disease Rating Scale (UPDRS-III) | Cf | SVM |
| Lu et al, 2020 | Surgery | Describe and predict regions of therapeutic tissue activation. | MER | Cf, R/TS | L1 LASSO, SVM |
| LeMoyne et al, 2020 | Tuning | Study of deep brain stimulation amplitude parameters. | Wearables | Cf | NN, Multilayer Perceptron |
| Mohammed et al, 2020 | Tuning | Present an approach that uses machine learning models to estimate the symptom severity of patients and adjust therapy accordingly. | LFPs | Cf, Ct | SVM, GMM |

**TABLE** **S3.** Publications included in the review under the category “Detection/management of symptoms”.

| **Detection/management of symptoms** | | | | | |
| --- | --- | --- | --- | --- | --- |
| **Author & Year** | **Stage** | **Goal** | **Input/Type of data** | **Model Group** | **Model used** |
| Liu et al, 2022 | Planning/Tuning | Describe a synchronized intracranial electroencephalogram recording and motion capture system. | EEG, Codamotion System. | Cf | RF |
| Oliveira et al, 2018 | Planning | Discriminating neurologically healthy individuals from those suffering from PD (treated with levodopa and DBS). | Wearables & EMG | Cf, DR/FS | SVM, PCA, Sammon's mapping, t-SNE |
| Kuhner et al, 2017 | Release | Extract global motor performance measures covering different everyday motor tasks as a function of a clinical intervention. | Wearables | R/TS, Cf | RF |
| Kuhner et al, 2020 | Release | Distinguish between healthy and PD subjects with DBS STN ON / OFF by identifying motion features and develop biomarkers for detecting and monitoring PD patients' motor symptoms. | 3D gait analysis | Cf | Weak classifier, Meta-Classifier AdaBoost |
| Angeles et al, 2017 | Tuning | Present a sensor system that can quantify the three cardinal motor symptoms of PD - rigidity, bradykinesia, and tremor. | Wearables | Cf | DT, SVM, KNN |
| Braga et al, 2019 | Tuning | Detect early signs of PD through free speech in uncontrolled background conditions. | Speech recordings | Cf | RF, SVM, NN |
| Chen et al, 2019 | Tuning | Investigate the sleep-stage classification based on LFPs in STN. | LFPs | Cf | SVM, DT |
| Geraedts et al, 2021 | Tuning | Develop an automated machine learning model based on preoperative EEG data to predict cognitive deterioration 1 year after STN DBS. | qEEG | Cf | RF w/ Bayesian optimization |
| Habets et al, 2020 | Tuning | Development of at machine learning logistic regression prediction model to generate probabilities for experiencing week motor response one year after surgery. | Preoperative variables | R/TS | LR |
| Hirschmann et al, 2022 | Tuning | To predict motor symptoms improvement from STN power and subthalamo-cortical coherence. | MEG, LFPs | Cf | Extreme-gradient Boosted Trees |
| Huo et al, 2020 | Tuning | Present a new wearable device for Parkinson’s diagnosis. | Wearables | Ct, Cf | 1-NN, Multilayer Perceptron, AdaBoost classifier |
| Khobragade et al, 2015 | Tuning | Apply the LAMSTAR (Large memory storage and retrieval) neural network for prediction of onset of tremor in PD patients to allow for on-off adaptive control of DBS. | Surface EMG, wearables | Cf | NN |
| Khodakarami et al, 2019 | Tuning | Ascertain whether an ambulatory wearable device could predict the response to levodopa from the response to the first morning dose. | Wearables | R/TS, Cf | LR, SVM, RBF kernel, Gradient Boosting DT |
| Kleinholdermann et al, 2023 | Tuning | Assess the benefits of noninvasive movement recordings as a means to predict best DBS settings. | Wearables | R/TS | RF |
| Livi et al, 2016 | Tuning | Discrimination and characterization of Parkinsonian rest tremors. | Laser based tremor frequency measurement | DR/FS, Cf | SVM |
| Liu et al, 2021 | Tuning | Investigate whether substantia nigra susceptibility features derived from radiomics with machine learning can predict motor outcome of STN-DBS in PD. | MRI | R/TS | LR |
| Mohammed et al, 2015 | Tuning | Create a patient customized detector for PD. | LFPs | Cf | SVM |
| Park et al, 2021 | Tuning | Predict motor function improvement by applying DL techniques to MER. | MER | Cf | DL |
| Peralta et al, 2021 | Tuning | Propose a machine learning-based method able to predict a large number of DBS clinical outcomes for PD. | Clinical data, MRI | Cf | ANN, SVM |
| Shah et al, 2018 | Tuning | Investigate the use of a logistic regression-based classifier to identify periods when PD patients have rest tremor by exploiting Local Field Potentials recorded with DBS STN. | LFPs | R/TS | LR |
| Shamir et al, 2015 | Tuning | Implementation of a clinical decision support system that incorporates patient-specific details on both stimulation and medication. | Clinical Data | Cf | SVM, NB, RF |
| Tahafchi et al, 2017 | Tuning | Propose of a FoG-detection method that captures temporal, spatial, and physiological features and uses a SVM to classify freezing episodes. | Wearables & EMG | Cf | SVM |
| Tracy et al, 2020 | Tuning | Explore voice for early detection of PD and its potential as a deep phenotype for PD. | Voice recordings | R/TS | L2 Ridge regularized logistic regression, random forest, gradient boosted decision trees. |
| Yao et al, 2020 | Tuning | Detect resting episodes in PD. | LFPs | Cf, R/TS, Ct, DR/FS | Kalman filtering, LR, SVM, LDA, Multilayer Perceptron, KNN, Extreme-gradient Boosted Trees, RF |

**TABLE S4**. Publications included in the review under the category “Identification of Biomarkers”.

| **Identification of Biomarkers** | | | | | |
| --- | --- | --- | --- | --- | --- |
| **Author & Year** | **Stage** | **Goal** | **Input/Type of data** | **Model Group** | **Model used** |
| Geraedts et al, 2021 | Tuning | Explore electroencephalography signals as biomarker of cognition using a Machine Learning pipeline. | EEG | R/TS | RF |
| Hirschmann et al, 2017 | Tuning | Tremor detection based on LFP based biomarkers. | LFPs | Cf | HMM |
| Khojandi et al, 2017 | Tuning | Develop a computational model which stratifies patients based on symptomatology into different frequency settings. | Preoperative MDS-Unified Parkinson’s Disease Rating Scale III scores. | Cf | RF, SVM, NN |
| Wang 2022 | Tuning | Comparison of resting state cortical connectivity between the off and off stimulation states and to healthy controls. | MDS-Unified Parkinson’s Disease Rating Scale, LFPs | Cf | RF, SVM |
| Zhang et al, 2020 | Tuning | To extract digital biomarkers of PD from crowd-sourced movement records. | Wearables | Cf, DR/FS | Deep CNN |

**TABLE S5**. Publications included in the review under the category “Peri-operative management”.

| **Peri-operative management** | | | | | |
| --- | --- | --- | --- | --- | --- |
| **Author & Year** | **Stage** | **Goal** | **Input/Type of data** | **Model Group** | **Model used** |
| Baumgarten et al, 2016 | Release | Propose of a pre-operative predictive model of PTSE. | Clinical data, MRI, CT | Cf | ANN |
| Chen et al, 2021 | Release | Investigate the association between brain morphology and initial STN-DBS efficacy. | CT, LFPs, MRI | Cf | SVM |
| Cao et al, 2019 | Surgery | Online identification of functional regions in deep brain stimulation based on an unsupervised random forest with feature selection. | MER | R/TS | RF |
| Hosny et al, 2021 | Surgery | Delineation of the neurophysiological borders of the STN along the electrode trajectory developed. | MER | Cf | CNN |
| Kim et al, 2019 | Surgery | Automatic localization of the subthalamic nucleus on patient specific clinical MRI. | MRI | R/TS | Regression |
| Khosravi et al, 2020 | Surgery | Presents an objective approach to help the surgical team in localizing the STN in real-time. | MER | Cf, R/TS | SVM, LR, KNN, DT, CNN |
| Ozturk et al, 2020 | Surgery | Compare the clinical benefit from SUA- versus LFP-based implantation using intraoperative online LFP processing. | LFPs | DR/FS | LDA |
| Valsky et al, 2017 | Surgery | Present machine learning classification procedures that utilize MER power spectra and allow for real time and high accuracy discrimination between STN and substantia nigra pars reticulata. | MER | Cf | SVM, HMM |
| Valsky et al, 2020 | Surgery | Propose a real-time machine learning classification of pallidal borders during deep brain stimulation surgery. | MER | Cf | HMM, L1 LASSO |
| Farrokhi et al, 202 | Tuning | Investigate preoperative clinical risk factors and predict adverse outcomes. | Clinical and demographic characteristics | Cf, R/TS | LR, XGBM, NN |

**TABLE S6**. Publications included in the review under the category “Signals decoding/encoding”.

| **Signals decoding/encoding** | | | | | |
| --- | --- | --- | --- | --- | --- |
| **Author & Year** | **Stage** | **Goal** | **Input/Type of data** | **Model Group** | **Model used** |
| Zhu et al, 2020 | Planning | Enable resource-efficient classification on a neural implant. | LFPs | Cf | Oblique DT |
| Camara et al, 2019 | Release | Study of the non-linear dynamical behaviour of STN-LFPs of Parkinsonian patients. | LFPs, EMGs | Cf | Recurrence Networks |
| Castaño-Candamil et al, 2020 | Tuning | Identifying controllable cortical neural markers. | Copy-drawn test , EEG | DR/FS, Cf, R/TS | LDA, source power co-modulation (SPoC), common spatial patterns (CSP) |
| Gilron et al, 2021 | Tuning / Release | Long-term wireless streaming of neural recordings for aDBS. | LFPs | Ct | Unsupervised clustering |
| Golshan et al, 2018 | Tuning | Present a human behaviour classification using LFP signals recorded from STN. | LFPs | Cf | SVM |
| Golshan et al, 2020 | Tuning | Classify human behaviour using the time-frequency representation of STN-LFPs within the beta frequency range. | LFPs | Cf | CNN |
| Islam et al, 2017 | Tuning | Explore deep brain LFPs for robust movement decoding of PD and Dystonia patients. | LFPs | Cf | NN |
| Khawaldeh et al, 2020 | Tuning | Test the hypothesis that increased synchronization denoted by beta bursting might compromise information coding capacity in basal ganglia networks. | LFPs | Cf | NB |
| Manum et al, 2015 | Tuning | Decode movement related behaviours from the recorded LFP activity. | LFPs | Cf | GNB, SVM |

**TABLE S7.** Division of Local Field Potentials (LFP) in the frequency bands described in literature. (STN – Subthalamic nucleus)

| **Band (range)** | **Description** |
| --- | --- |
| Delta bands  (0.3Hz) | Literature related to delta band is scarce. |
| Theta (4-7Hz) &  Alpha (8-12Hz) bands | STN low frequency (LF) oscillations have been studied in extensive non-motor domains. LF/Beta band power ratio has advantages over the single use of beta band power. |
| Beta bands  (13-35Hz) | Beta band is the most studied band as biomarker for “smart” DBS design. Suppression of beta band is associated with improvement of motor symptoms. |
| Gamma band  (31-200Hz) | The gamma band activity is seen as prokinetic, with a compensatory mechanism toward the akinetic role of beta activity. |
| High frequency oscillations  (above 200Hz) | High frequency oscillations are regarded as prokinetic, and STN activity increases at the onset of the movement and Levodopa administration. |


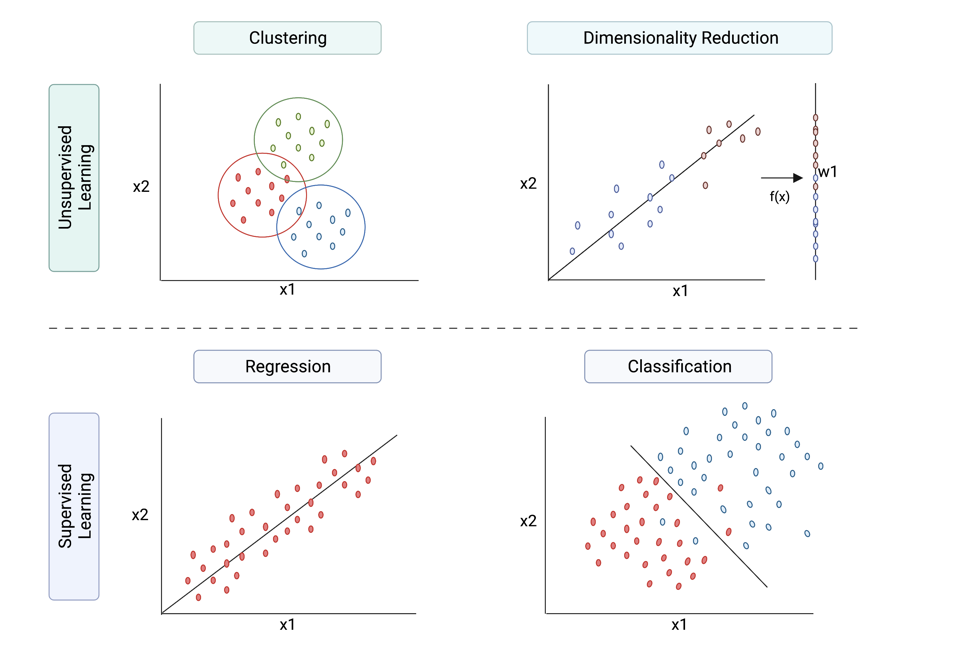


**Figure S1.** Visual representation of the mentioned areas of machine learning. The two main areas listed here are supervised and unsupervised learning (a third type is reinforcement learning, but is outside the scope of this paper). On the top row, two methods of unsupervised learning (methods that look for hidden patterns or internal structures in the input, without pre-defined information about the output) are represented: clustering (a method that groups sets of data according to similarities or goals), here presenting three possible clusters, and dimensionality reduction (methods that search for a reduced set of variables describing the data), here considering two initial dimensions collapsed in one output variable. The bottom row refers to supervised learning (models that are trained on labeled, or annotated, datasets), presenting regression (which produces continuous outputs), and classification (which are associated with categorical/discrete outputs), both considering here two input variables.


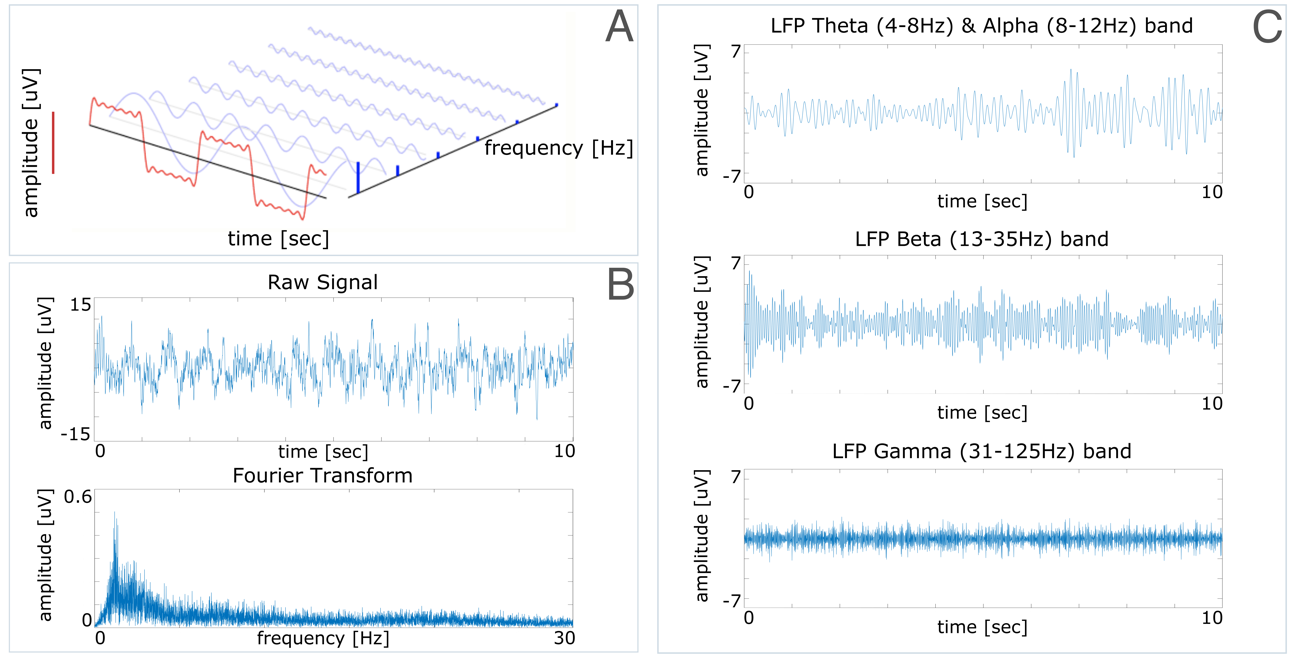


**Figure S2.** Signal decomposition in the frequency domain. (**A**) Decomposition of the time domain signal in its frequency components. (**B**) Raw signal and Fourier transform of Local Field Potentials (LFP). In the Fourier transform plot it is possible to identify the frequency composition of the signal. (**C**) Representation of three frequency bands of LFP signals: theta & alpha band (4-12Hz), beta band (13-35Hz) and gamma band (31-200Hz).

Panel A adapted from:

<https://tex.stackexchange.com/questions/127375/replicate-the-fourier-transform-time-frequency-domains-correspondence-illustrati>.


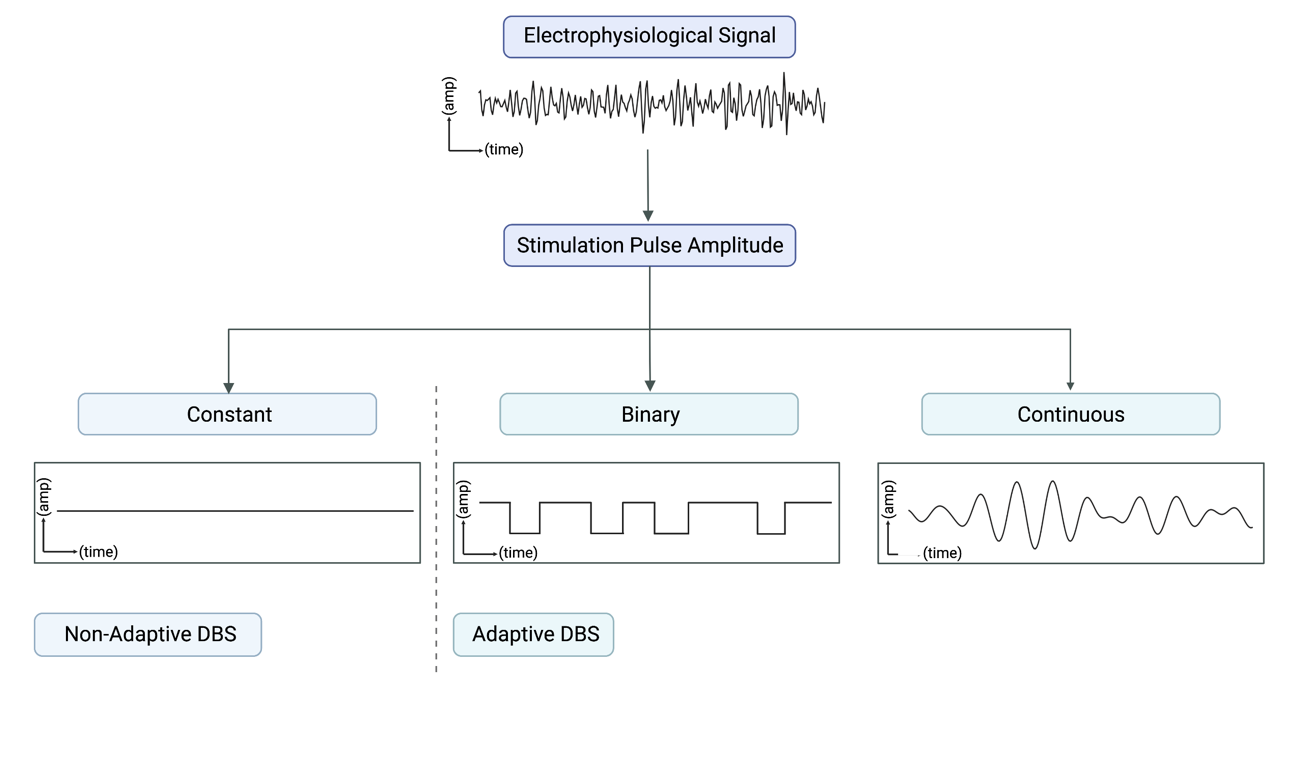


**Figure S3**. Types of control mechanisms in Deep Brain Stimulation using electrophysiological signals as input. Three standard control mechanisms for DBS: non-adaptive, adaptive with binary outputs, and adaptive with continuous outputs. Concerning an electrophysiological signal, the control system first reads the signal, and then decides on the stimulation pulse amplitude. In the case of non-adaptive DBS, this amplitude is constant, and it will be independent of the electrophysiological signal behavior. When considering adaptive DBS, the amplitude will vary with the readings of the electrophysiological signal. In the case of binary control, a threshold for stimulation is defined, and every time the electrophysiological signal crosses above the threshold, the stimulation is turned ON, and turned OFF when the signal shifts below the threshold. In the case of continuous control, the amplitude is being adapted to the continuous reading of the signal and varying according to this behavior.
